# Help! I need somebody: Development and validation of the Romantic Support-Seeking (RoSS) scale

**DOI:** 10.1007/s12144-025-08749-0

**Published:** 2025-12-22

**Authors:** Rhia E. Perks, Laura M. Vowels, Claire M. Hart, Rachel R. R. Francois-Walcott, Katherine B. Carnelley

**Affiliations:** 1 Centre of Research on Self and Identity (CRSI), School of Psychology, University of Southampton, Southampton, UK; 2 School of Psychology, University of Surrey, Guildford, UK; 3 FAmily and DevelOpment research center (FADO), Institute of Psychology, University of Lausanne, Lausanne, Switzerland; 4 School of Psychology, University of Roehampton, London, UK

**Keywords:** Support-seeking, Romantic relationships, Scale development, Validation, Thriving through relationships, Attachment

## Abstract

Research has established distinct categories of support-seeking behaviors, including direct, indirect, emotional, and instrumental support-seeking. However, no existing scale incorporates all four types of support-seeking within romantic relationships in one measure. Understanding how individuals seek support from romantic partners is crucial for managing stress, relationship satisfaction, and well-being. We aimed to create and validate the Romantic Support-Seeking (RoSS) scale, grounded in theory and empirical data. In Study 1 (*N* = 117 students), we used open-ended questions to gain knowledge on support-seeking behaviors and inform item development. In Study 2 (*N* = 491), we conducted an exploratory factor analysis to assess the factor structure and select the highest-loading items. In Study 3 (*N* = 355 students), we used confirmatory factor analysis to confirm the factor structure and provide preliminary construct validity evidence by correlating the subscales with measures of attachment, relationship quality, and coping. We identified four reliable subscales: direct emotional support-seeking; direct instrumental support-seeking; indirect support-seeking; and no support wanted. This accounts for each type of support-seeking, and individuals who choose to manage distress alone instead of seeking support. The RoSS is a significant advancement over existing measures because it captures the full spectrum of romantic support-seeking. The samples were predominantly young, White, and female so future work should address whether the scale applies to other demographic groups. This has clinical and research implications for understanding support dynamics in relationships and their links to individual and relational outcomes, which may be used in counselling to help couples navigate distress effectively.

**Supplementary Information:**

The online version contains supplementary material available at 10.1007/s12144-025-08749-0.

As The Beatles highlighted, people sometimes need help from close others to reduce distress. The theory of thriving through relationships underscores the importance of well-functioning close relationships, which provide support during both challenging times and opportunities in life (Feeney & Collins, 2015b). This is rooted in attachment theory, which states that people seek proximity to close others during distress and during exploration of the environment (Bowlby, 1969). Those in supportive relationships have higher relationship satisfaction (Hilpert et al., 2018). A mismatch between support desired and caregiving received is known as a support gap (for a review, see McLeod et al., 2020).

To receive this desired help, individuals may need to actively seek support from close relationships (Feeney & Collins, 2015a; Forest et al., 2021). This is known as support-seeking - an individual’s attempt to elicit help from a caregiving figure (Collins & Feeney, 2000). Support-seeking is governed by the attachment system and is crucial to survival in infancy but remains important throughout the life span to gain safety and reassurance during threats or opportunities (Bowlby, 1969). Support-seeking divides into direct and indirect forms. Direct support-seeking refers to explicit requests for support, whereas indirect support-seeking uses behavioral cues or hints (Fraley & Shaver, 1998) – like sulking or whining (Don et al., 2019). Support itself can be divided into emotional and instrumental – an individual may specifically seek one type of support. Emotional support describes reassurance and affection whilst instrumental support refers to practical, tangible assistance (House, 1981).

Direct support-seeking is used frequently during stress as partners are used as a Safe Haven, leading to helpful caregiving (Collins & Feeney, 2000; Feeney & Collins, 2015a; Wang et al., 2023). Caregiving is associated with higher relationship satisfaction and buffers against stress (Hilpert et al., 2018). Direct support-seeking is commonly used by individuals with a secure attachment style (Collins & Feeney, 2000; Mikulincer & Shaver, 2016). Contrastingly, indirect support-seeking is usually fostered by individuals high in attachment insecurity (Mikulincer & Shaver, 2016), or due to fear of stigma or rejection (Collins & Feeney, 2000; Don et al., 2013; Williams & Mickelson, 2008). Indirect support-seeking leads to ineffective caregiving from partners (Don et al., 2013; Williams & Mickelson, 2008) and negative perceptions of partner support, which negatively predicts relationship satisfaction (Don et al., 2013). For a review, see Wang et al. (2023).

Research separates emotional and instrumental support (e.g., Mikulincer & Florian, 1997; Rife et al., 2016) and individuals may seek either type, depending on the situation and personal preference. Typically, individuals seek emotional support for social stressors (Rife et al., 2016) and instrumental support for goal-related stressors (Chan, 2013). Securely attached individuals, compared to insecure, seek more emotional and instrumental support (Florian et al., 1995). Avoidantly attached individuals prefer instrumental support (Mikulincer & Florian, 1997) whereas anxiously attached individuals favour emotional support (Shaver et al., 2005) - instrumental support can exasperate their distress (Mikulincer & Florian, 1997). Receiving the desired type of support is most beneficial and this is elicited by direct support-seeking (Cutrona et al., 2007).

However, it is important to note that not all individuals actively engage in support-seeking. For some, this reflects a desire for support but a hesitation to seek support. Indeed, the amount of support desired often exceeds the amount of support sought, which in turn means less support is received than desired (High & Crowley, 2018). This can be true for those facing stigma (High & Crowley, 2018) or fearing rejection (for a review, see Wang et al., 2023). However, some individuals do not want support. In fact, receiving more support than desired is associated with negative outcomes (High & Crowley, 2018; McLeod et al., 2020) – particularly when the support provided is not responsive to the attachment style of the support recipient (for a review, see McLeod et al., 2020). Those with an avoidant attachment typically favour independence and self-reliance so do not want support and therefore engage in less support-seeking (see Mikulincer & Shaver, 2016). This goes beyond reluctance to seek support and instead reflects an active desire to cope and handle stress independently.

Despite clear distinction of these categories of support-seeking, or desire not to seek support, no existing scale encompasses these constructs. Herein we describe our development of a self-report scale that assesses these distinct strategies within romantic relationships. We report on the scale’s reliability and validity across three varied samples. Because self-report is commonly used in psychology (Haeffel & Howard, 2010), this scale will fill the gap for a reliable, valid measure of different types of support-seeking within romantic relationships – an important construct to assess considering the associated individual and relational outcomes. This will benefit researchers and practitioners by providing a standardized tool to identify effective and ineffective support-seeking techniques for coping with distress, enabling comparisons across studies, and can be used by clinical and counseling practitioners to improve couples’ ability to seek and receive the support they need, ultimately enhancing their well-being and relationship quality.

## Existing measures

Previous self-report measures to assess support-seeking are limited. Relevant scales include the Dyadic Coping Inventory (DCI), the Coping Orientation to Problem Solving Inventory (COPE), the Berlin Social Support Scale (BSSS), and the Support-Seeking Strategy Scale (SSSS) – most of these do not solely focus on support-seeking and some are not relevant or applicable to the context of romantic relationships.

The DCI (Bodenmann et al., 2008) is widely used, with validated versions in many countries and language. This is specifically for use in romantic contexts. In particular, the Stress Communicated by Oneself (SCO) subscale asks participants to rate support-seeking frequency, but does not distinguish emotional, instrumental, direct, or indirect forms. Additionally, it does not account for individuals who have an active preference to handle distress alone. As a result, low SCO scores do not distinguish between reluctance to seek support and a deliberate preference for self-reliance in coping with stress.

The COPE (Carver et al., 1989) includes two relevant subscales that ask participants to rate frequency of *use of emotional support* (e.g., “I get sympathy and understanding from someone”) and *use of instrumental support* (“I talk to someone who could do something more concrete about the problem”). Notably, some of these items focus on utilisation of support rather than support-seeking and does not distinguish direct or indirect support-seeking, nor does it account for individuals who do not want support. These subscales are not specific to romantic support-seeking, but some researchers adapt the items for this purpose (e.g., Tement et al., 2023).

The BSSS (Schulz & Schwarzer, 2003) includes a subscale measuring support-seeking (e.g., “Whenever I am down, I look for someone to cheer me up again.”) but does not distinguish between types of support-seeking. Some items focus more on the general act of seeking support rather than the specific behaviors and strategies used to seek support. No items assess indirect support-seeking, nor do any items account for individuals who do not want support. Resultantly, low BSSS scores do not differentiate between reluctance to seek support and an active desire to handle stress independently. This scale was created for cancer healthcare settings (Schulz & Schwarzer, 2003), so is not relevant to romantic relationships because it does not capture the unique dynamics and strategies involved in support-seeking between partners.

The SSSS (Marshall et al., 2023) consists of three subscales: direct support-seeking (e.g., “I voice my needs directly”), hyperactivation (e.g., “I make repeated requests for help”), and deactivation (e.g., “I tend to downplay my needs”). These do not distinguish between types of support. An indirect subscale was hypothesised but not included in the final items so the construct cannot be measured. Furthermore, the scale was developed to measure older adult’s support-seeking from their adult children. Thus, no existing scale allows researchers to measure the distinct categories of support-seeking within one measure. Moreover, many of these scales are not relevant to romantic relationships.

Both the SCO and COPE assess frequency of self-reported behaviors, whereas the BSSS and SSSS use agreement-based items. Frequency-based items better capture responses to specific events, whereas agreement-based items better capture extreme scores and subjective content related to general attitudes and behaviors (Marfeo et al., 2014). Because behaviors fluctuate over time, it may be beneficial for participants to rate general support-seeking behaviors rather than time frames. The use of different scales and items makes it difficult to compare results across studies. A scale assessing these unique strategies within one coherent measure will aid comparison across the literature. In addition to self-reports, researchers utilise behavioral measures of support-seeking –like behavioral observations (e.g., Collins & Feeney, 2000; Fraley & Shaver, 1998; Mikulincer & Florian, 1997). Behavioral categories of direct support-seeking include asking for help, whereas indirect support-seeking includes hinting, complaining, sulking (Collins & Feeney, 2000), acting weak, and debasing the self (Don et al., 2019). The distinction between emotional and instrumental support within observational studies generally focuses on support provision, rather than support-seeking. The social support interaction coding system (Pasch et al., 2004) is used to separate support behaviors into observational categories (e.g., Verhofstadt et al., 2007) - emotional support includes providing reassurance and sympathy, whereas instrumental support includes offering assistance and advice (Pasch et al., 2004).

Naturalistic observation and behavioral measures are not always possible, so researchers need a reliable self-report measure of support-seeking. Self-report is widely used in psychology (Haeffel & Howard, 2010) due to its practicality and cost-efficiency (Paulhus & Vazire, 2009). Support behaviors may be too subtle to detect within one interaction, so participants are more likely to perceive these differences when considering support generally (Verhofstadt et al., 2007). However, many social support studies use non-validated measures or develop their own instruments (for a systematic review, see McLeod et al., 2020) – this does not ensure validity and reliability, and makes it challenging to directly compare findings across studies. Thus, it is necessary to establish a validated measure of support-seeking in romantic relationships.

## The current research

This research aimed to create a reliable, valid scale to measure different types of support-seeking, or preference not to seek support, from romantic partners. Initial items were created and tested across three studies. In Study 1, we used open-ended questions to identify support-seeking behaviors used within relationships. In Study 2, we tested an initial pool of 34 items using exploratory factor analysis. In Study 3, we used confirmatory factor analysis to validate the factor structure and provided preliminary construct validity by correlating subscales with relevant measures – including the SCO, because this is the most relevant existing scale. We did not correlate with the COPE as this focuses on support utilisation, or the BSSS and SSSS which are not relevant to romantic relationships. All data and results are available on Open Science Framework (https://osf.io/rv56c/?view_only=83c4a6489a2b46698ed68a71ec230fd7).

## Study 1

Qualitative research can enrich the quality of research by adding a useful empirical basis for scale development and maintaining focus on human experience (Rowan & Wulff, 2007). By conducting qualitative research before item development, in-depth information can be obtained from participants concerning the actual support-seeking behaviors they use or recommend. We used two qualitative methods to inform item development. Prior to Study 1, we conducted qualitative interviews in which participants discussed support-seeking. Several support-seeking behaviors were identified within themes of direct support-seeking, indirect support-seeking, and no support-seeking. These behaviors informed item development. Most participants noted that they directly requested support from their partner when needed and this benefitted their relationship. Some participants utilised indirect support-seeking by expressing distress through tone of voice, physical expression and behaviors, or partner’s intuition. Other participants specified that they did not need support, or actively avoided support. Complete findings can be found in Francois-Walcott et al. (2024). Next, Study 1 utilised hypothetical vignettes and open-ended questions regarding support-seeking. The interviews allowed participants to freely describe support-seeking, whereas Study 1 provided specific scenarios regarding goals to encourage thoughts regarding types of support-seeking. Vignettes have demonstrated high validity and generalise to ‘real-life’ behaviors (Evans et al., 2015) so provide insight into support-seeking behaviors.

### Method

#### Participants

We expected a sample of 100 people to provide sufficient variety in responses to cover a range of support-seeking behaviors. A total of 162 participants began the study, but data was removed for anyone who did not complete the whole survey (*n =* 45). A total of 117 participants were included in the analysis. Participants were required to be at least 18 years old and currently be in a relationship of at least 6 months. On average, participants were 19.70 years old (*SD* = 1.64, range 18–28). Participants were primarily women (91.45%). Most participants were in a romantic relationship (82.90%), dating (13.67%), or cohabiting (3.42%) with an average relationship length of 1.83 years (*SD =* 1.22). Participants identified as heterosexual individuals (89.08%), bisexual individuals (10.08%), or lesbian/gay individuals (0.84%). Participants were White (83.76%), Asian (6.72%), Mixed race (6.72%), and Black (5.04%).

#### Procedure

Ethical approval was obtained from the institutional review board. Students from a UK university were recruited and received course credits as compensation. The survey was hosted on Qualtrics and conducted from March to May 2019.

Participants provided demographic information before being presented with three vignettes created for this study. These vignettes described hypothetical scenarios regarding their own relationship, five years into the future. Aspects of the vignettes were manipulated to experimentally assess whether contextual factors influenced participant’s views on support-seeking. All participants were presented with the same vignette in segment one, in which one partner received an opportunity:

*Imagine that you have graduated and have been together with your current partner for at least five years*,* live together*,* and are committed to the relationship. You are both working in jobs that are relevant to what you wanted to do after graduation. Your partner gets an opportunity for a promotion at work. Your partner comes to tell you about the opportunity and wants to discuss whether to take the promotion or not.*

For segments two and three, participants were randomly allocated to receive one of the following scenarios. They either received the opportunity themselves in segment two and their partner received the opportunity in segment three, or vice versa.

Segment two: *Your partner tells you that the promotion would mean a salary increase with additional responsibilities but “would not take any more time than their current role” OR “would mean working significantly longer hours” OR “would involve you both relocating to another country”.*

Segment three: *“You decide together that the opportunity is too good to pass up and your partner takes the promotion” OR “You decide together that it is not the right time to take the promotion”. A year later*,* you get an opportunity to study a postgraduate course which could enhance your career prospects in the long-term and “you can study alongside your current job” OR “it would mean that you would need to attend weekend seminars alongside your job and would have less time to spend together with your partner” OR “it would mean that you would have to relocate to another city and live apart for two years whilst you complete the course”.*

Following each vignette, participants were asked “*What could you do to show that you need support from them for the opportunity?”* or *“What could your partner do to show that they need support from you for the opportunity?”*, depending on the vignette they received. Content analysis was used to analyze the data, coded by two research assistants. Cohen’s Kappa ranged between 0.82 and 1.00 for the responses. Any discrepancies were settled by the second author. Codes were created using a deductive approach, based on previous research separating direct and indirect support-seeking (Fraley & Shaver, 1998), with inductive additions for new categories as needed.

### Results

Four themes were identified within support-seeking: *direct*,* indirect*,* relationship-focused*, and *no support-seeking.* Within these themes, 11 subthemes were identified (see Table 1). Both *direct* and *indirect* had been determined using the deductive approach based on previous literature. Both *relationship-focused* and *no support wanted* were added using the inductive approach to reflect behaviors that did not fit into existing codes.

**Table 1:** Frequencies of themes and subthemes discussed by participants in Study 1

Theme	Subtheme	Segment 1	Segment 2		Segment 3
Direct	Ask directly	96	56		75
	Discuss feelings	40	30		26
	Discuss options	85	60		66
	Seek reassurance	1	5		4
Indirect	Hint/complain	2	0		1
	Talk up the opportunity	11	11		16
	Speak to other people	3	0		0
	Talk about the opportunity	1	0		0
Relationship-focused	Provide reassurance	21	23		17
	Involve partner in goal	6	10	4	
	Address impact on relationship	2	21	15	
No support seeking		7	5	6	

#### Direct

*Direct* support-seeking was discussed by most participants and consisted of four subthemes. Firstly, *ask directly* – e.g., “ask [their partner] what [they] would do and what advice [they] have”. Second, *discuss feelings* –e.g., “clearly explain how they’re feeling and ask for the same in return”. Third, *discuss options* – e.g., “have a discussion about the pros and cons”. Lastly, *seek reassurance* – e.g., “be upfront about what kind of reassurance and support [they want]”.

#### Indirect

Some participants discussed *indirect* support-seeking, which consisted of four subthemes. First, *hint/complain* – e.g., “remind [their partner] that a relationship is about sacrifice”. Second, *talk up opportunities* – e.g., “talk about the benefits… and possibly any benefits for them both as a couple”. Third, *speak to other people* outside of the relationship – e.g., “get an outsider’s view on the situation”. Lastly, *talk about opportunities* – e.g., “tell [their partner] about the details”.

#### Relationship-focused

*Relationship-focused* was created inductively because it did not fit either *direct* or *indirect*, because it did not necessarily involve support-seeking. Rather, it involves ensuring both partners feel involved and supported, so still provides valuable insight into romantic support. This consisted of three themes. First, *provide reassurance* – e.g., “stress how the relationship is still [the] top priority”. Second, *involve partner in goal* – e.g., “make them feel involved rather than forgotten”. Third, *address impact on relationship* – e.g., “discuss any problems [that] it may cause in their relationship.

#### No support-seeking

Lastly, *no support-seeking* was created inductively because it did not fit *direct* or *indirect* – contrastingly, participants specified that they would not seek support. Some participants believed “they shouldn’t have to be dependent on partner, [they] can make [an] independent decision” so did not want support at all, Alternatively, other participants felt support-seeking was not necessary because support “should be there already” without needing to ask for it.

### Discussion

Study 1 revealed that individuals seek support directly and indirectly from their partner. Direct support-seeking included desire for emotional reassurance, or practical assistance in decision-making. Similarly, participants discussed providing reassurance towards a partner in relationship-focused support behaviors, to ensure both partners feel involved and supported. Contrastingly, indirect support-seeking used cues and hints to elicit support without asking directly. Importantly, other participants noted that they would not engage in support-seeking for one of two reasons, First, some participants actively preferred independence and so did not want support from their partner. Other participants believed support should be present without needing to ask for it. However, the vignettes were hypothetical and may not capture the complexity of real-life relationships. Additionally, the vignettes focused on support-seeking regarding goals so may not generalise to other support-seeking situations. Therefore, in Study 2, we developed an initial pool of items based on Study 1 and the previous interview study addressing general support-seeking.

## Study 2

The themes identified within Study 1 informed the creation of potential items to measure support-seeking within Study 2. We aimed for broad applicability by creating items focused varied support-seeking strategies **-** including direct, indirect, instrumental, and emotional. Study 1 identified a distinction between participants who did not want to seek support but still expected to receive support, and participants who actively preferred to handle distress alone. Therefore, we ensured some items covered an active preference to not receive support, compared to a reluctance to seek support which would be reflected in low scores on other subscales. We conducted an exploratory factor analysis (EFA) to assess the factor structure of the scale and identify the most effective items for measuring each factor. We expected to find direct support-seeking, indirect support-seeking, and no support wanted. We anticipated that direct support-seeking may divide into emotional and instrumental, in line with Study 1 and previous research, whereas indirect support-seeking is generally ambiguous so this distinction is not evident. The study had a relatively large sample to aid generalisability, reliability, and validity.

### Method

#### Item pool development

An initial item pool of 56-items was generated by the authors based on the interview study presented elsewhere (Francois-Walcott et al., 2024), Study 1 findings, theoretical understanding, and a review of existing support-seeking measures (Bodenmann, 2008; Carver et al., 1989; Schulz & Schwarzer, 2003). We removed items which we felt were not relevant to our framework – for example, “I’m open to my partner helping me with opportunities” did not specifically demonstrate support-seeking. We removed or combined overlapping items (e.g., “I seek reassurance or comfort from my partner” and “I seek emotional comfort and reassurance from my partner”). A total of 34-items remained. These items asked participants to indicate their agreement from 0 (*disagree strongly*) to 10 (*agree strongly*); sample item: “I seek sympathy and understanding from my partner”. We used an 11-point Likert scale to increase sensitivity, better approximate normality, and clearly reflect a complete absence or presence of a behaviour (‘0’ and ‘10’; Leung, 2011) – in our data, the item-level skewness and kurtosis values were within acceptable ranges. Study 2 aimed to examine the factor structure of the scale and determine the most relevant items - to reduce the number of items and ensure the final scale consisted of parsimonious, functional, and internally consistent items (Boateng et al., 2018) while reducing respondent fatigue (Sinickas, 2007).

#### Participants

Participants were required to be at least 18 years old and currently in a romantic relationship of at least 6 months. Participants were recruited via social media (*n =* 411; e.g., Twitter) and Prolific (*n =* 232) and took part in an online survey on Qualtrics between 3rd December 2020 and 24th February 2021. Participants recruited via Prolific received £1.00 for a 10-minute survey, while social media participants received no incentive. A total of 643 participants began the study, but data was removed for participants who did not meet the eligibility requirements (*n =* 48) or did not complete the entire support-seeking measure (*n =* 106). A total of 491 participants were included in the analysis, aligning with research suggesting that factor analysis requires 300 participants, or ratios of 5:1 or 10:1 participants per item (for a review, see Boateng et al., 2018).

Participants were primarily living in the United Kingdom (38.76%). Most participants were women (67.3%) with an average age of 27.36 years (*SD =* 9.54, range 18–71). Most participants identified as White (78.91%), Asian (3.61%), Black (1.61%), or Mixed race (1.00%). Participants primarily identified as heterosexual individuals (79.91%), bisexual individuals (13.45%), and lesbian or gay individuals (3.41%). The majority were in a committed relationship (53.21%), dating (8.83%), cohabiting (15.26%), and married (22.69%) with an average relationship length of 5.58 years (*SD =* 7.00) Most relationships were monogamous (94.58%) and most participants did not have children (82.12%).

#### Procedure

Participants were presented with an information sheet, then answered questions requesting demographic information. They were given the 34-item support-seeking measure and the following prompt:

*When you’re feeling distressed and upset and you need support from your partner*,* what do you do? Please indicate the extent to which you agree with each statement from 0* (disagree strongly) – *10* (agree strongly).

All participants completed scales measuring attachment (Lafontaine et al., 2016), relationship quality (Fletcher et al., 2000), perceived support, perceived stress (Cohen et al., 1983), life satisfaction (Diener et al., 1985), goal outcomes, dyadic coping (Bodenmann et al., 2008), and COVID-19 worry (Taylor et al., 2020) as part of a concurrent study - these were not included in the present analyses.

### Results

A Kaiser-Meyer-Olkin test revealed a sampling adequacy of 0.92 – above the recommended value of ≥ 0.70; (Watkins, 2018). Bartlett’s test of sphericity demonstrated that the data were appropriate for factor analysis, χ^2^ (34.03) = 25362.20, *p <*.001. Additionally, the skewness was below +/**-** 2 and kurtosis below +/**-** 7 (Curran et al., 1996) for each item. Thus, the data was analyzed using EFA in R 4.2.2 (R Core Team, 2022). We used Maximum Likelihood extraction with Oblimin rotation to allow for correlation between the factors. We used three indicators to determine the number of factors to extract: Kaiser’s rule that eigenvalues should be greater than 1; scree plots of the eigenvalues; and parallel analysis using nFactors (Raiche & Magis, 2020) and lavaan (Rosseel, 2012). We retained items that loaded > 0.50.

The EFA revealed five factors, with no cross-loadings (see Table 2): *direct emotional support-seeking; direct instrumental support-seeking; indirect support-seeking; no support wanted;* and *partner just knows support is needed*. The scale explained 47% of variation. We also considered the qualitative content validity of these items and factors (Goetz et al., 2013; Stanton et al., 2006) and chose to remove *partner just knows support is needed* because it did not reflect support-seeking behaviors so did not fit within the scale. Conceptually, this seems to differ from the other factors by reflecting passive support dynamics rather than active support-seeking. The unique variance explained for each factor was also calculated. *Direct emotional support-seeking* accounted for 28.78% of the overall variance explained by the scale. *Direct instrumental support-seeking* accounted for 28.20% of the variance explained. *Indirect support-seeking* explained 21.18% of the variance. *No support wanted* accounted for 21.84% of the variance. The highest loading items were selected for each factor – 17 items were retained, loading < 0.50 (ranging from 0.51 − 0.79). Four items each were selected for *direct instrumental support-seeking* (*ω =* 0.89, *ωH =* 0.79, *α* = 0.86), *indirect support-seeking* (*ω =* 0.80, *ωH =* 0.71, *α* = 0.75), and *no support wanted* (*ω =* 0.79, *ωH =* 0.74, *α* = 0.77), and five items for *direct emotional support-seeking* (*ω =* 0.85, *ωH =* 0.81, *α* = 0.83).

**Table 2:** Subscales identified from the EFA*,* item loadings for each factor from both EFAs, and descriptive Statistics

Item	Direct emotional (EFA 1/2)	Direct instrumental (EFA 1/2)	Indirect (EFA1/2)	No support wanted (EFA 1/2)	Partner just knows (EFA 1)	M	SD	Sk	Ku
**I seek reassurance or comfort from my partner.**	**0.79/0.83**	0.02/0.00	− 0.04/− 0.04	− 0.01/− 0.02	0.03	7.73	2.27	−1.14	4.02
**I ask for physical comfort**,** such as hugs**,** from my partner.**	**0.65/0.67**	0.00/0.03	− 0.06/− 0.06	− 0.04/− 0.05	0.06	7.64	2.54	−1.07	3.47
**I seek verbal reassurance from my partner rather than advice (e.g.**,** “everything will be okay”).**	**0.61/0.59**	− 0.10/− 0.15	0.16/0.19	− 0.04/− 0.06	− 0.03	5.85	2.75	−0.35	2.30
**When I feel stressed**,** I go to my partner for encouragement.**	**0.57/0.59**	0.19/0.24	− 0.06/− 0.03	− 0.06/− 0.04	0.13	7.28	2.35	−0.94	3.52
**I seek sympathy and understanding from my partner.**	**0.53/0.60**	0.23/0.19	0.12/0.13	0.03/0.02	0.09	7.46	2.41	−1.11	3.96
If I am upset or need support I often cry.	0.50	− 0.15	0.25	− 0.01	− 0.09	5.23	3.53	−0.06	1.56
I discuss my feelings with my partner.	0.43	0.42	− 0.05	− 0.10	− 0.03	7.80	2.33	−1.32	4.48
When I am worried, I reach out to my partner to talk to.	0.39	0.32	− 0.04	−0.15	0.11	7.65	2.24	−1.12	4.12
I vent at my partner and they will know I need support.	0.32	0.17	0.29	− 0.01	0.16	5.70	2.94	−0.40	2.16
I speak to people other than my partner about the situation.	0.18	0.15	0.12	0.14	− 0.26	5.11	2.99	−0.12	1.92
**I ask to discuss different options with my partner.**	− 0.09/− 0.07	**0.75/0.84**	0.06/0.09	0.01/0.03	0.18	7.11	2.35	−0.92	3.53
**I discuss the pros and cons about the situation with my partner.**	0.14/0.10	**0.66/0.74**	− 0.09/− 0.06	0.07/0.08	0.10	7.25	2.43	−0.96	3.36
**I ask my partner for advice directly.**	0.10/0.10	**0.65/0.67**	− 0.05/− 0.05	− 0.15/− 0.14	0.11	7.14	2.48	−0.85	3.18
**I ask my partner for help directly.**	0.20/0.09	**0.64/0.59**	− 0.03/− 0.01	− 0.19/− 0.21	− 0.10	7.00	2.49	−0.75	2.97
Whenever I need help, I ask my partner for it.	0.25	0.38	0.05	− 0.22	0.10	6.66	2.40	−0.63	2.94
I ask my partner what they have done in similar circumstances.	0.11	0.37	0.06	− 0.09	0.24	6.26	2.77	−0.59	2.56
If I do not know how to handle a situation, I ask my partner what they would do.	0.11	0.36	0.05	− 0.12	0.33	6.76	2.56	−0.85	3.17
I tell my partner I just need to vent out my frustration and ask them to listen.	0.31	0.32	0.18	0.01	− 0.08	5.72	2.81	−0.33	2.23
**I complain to my partner that they haven’t been supportive toward me.**	0.01/0.01	0.07/0.06	**0.76/0.74**	− 0.07/− 0.01	− 0.09	3.36	2.98	0.65	2.27
**I shout or yell at my partner if they are not supporting me.**	− 0.10/− 0.12	0.02/0.00	**0.69/0.74**	− 0.05/− 0.05	− 0.08	1.91	2.53	1.45	4.27
**I sulk if I feel unsupported by my partner.**	0.17/0.23	− 0.08/− 0.06	**0.61/0.57**	0.02/0.08	0.01	4.52	3.06	0.12	1.90
**I provide non-verbal cues such as huffing and puffing.**	− 0.02/0.12	− 0.04/− 0.01	**0.54/0.50**	− 0.03/0.07	0.27	4.38	3.03	0.11	1.87
I may use hints to get my partner to support me.	− 0.14	− 0.07	0.50	0.13	0.23	4.87	2.81	−0.11	2.06
I wait for my partner to see the signs that I need support.	0.06	− 0.16	0.43	0.27	0.24	4.01	2.70	0.23	2.11
**I prefer to do things myself rather than ask my partner for support.**	0.02/0.02	− 0.03/− 0.01	− 0.07/− 0.03	**0.79/0.83**	0.04	4.80	2.68	0.04	2.17
**I prefer to solve my problems by myself rather than ask my partner.**	− 0.03/− 0.04	0.03/0.01	− 0.07/− 0.01	**0.76/0.73**	− 0.06	4.94	2.63	−0.04	2.23
**I don’t tell my partner if I’m stressed unless it’s absolutely necessary.**	− 0.11/0.09	− 0.07/− 0.04	− 0.04/0.00	**0.58/0.57**	0.05	4.16	3.08	0.31	1.85
**I have a hard time accepting support from my partner even if they offer.**	0.02/0.02	− 0.04/− 0.06	0.23/0.24	**0.51/0.46**	− 0.01	2.79	2.63	0.93	2.96
I struggle to directly ask for help.	0.06	− 0.26	0.06	0.42	0.14	4.59	3.08	0.08	1.83
I go quiet or stop responding to my partner when I am struggling.	0.05	− 0.10	0.30	0.39	0.16	4.57	2.94	0.05	1.88
I seek space from my partner.	− 0.16	0.38	0.27	0.39	− 0.22	4.07	2.78	0.31	2.26
My partner can tell I need support by my facial expressions.	0.05	0.06	0.04	− 0.05	0.72	6.88	2.59	−0.94	3.19
It is obvious to my partner when I am struggling with a task.	0.06	0.12	0.04	0.02	0.60	6.36	2.50	−0.58	2.79
I don’t need to ask for support, my partner just knows.	− 0.01	0.11	− 0.12	− 0.04	0.57	5.53	2.74	−0.30	2.24

After reducing the factors and items, we ran the EFA again (see Table 2). The model explained 53% of the variation. Each item loaded > 0.50, except for one item from the *no support wanted* subscale (“I have a hard time accepting support from my partner even if they offer”), which had a factor loading of 0.46. Much research utilises a factor loading cut off below 0.45 (for a review, see Howard, 2016), so we chose to retain this item. Additionally, we considered the qualitative content of this item (Goetz et al., 2013; Stanton et al., 2006), which aligns with the other items on the subscale while providing enough differentiation that a broader range of behaviors are captured.

An independent samples t-test was conducted to compare the support-seeking behaviors between participants recruited via Prolific and via social media, using the four subscales and 17 items identified in the EFA. There was no significant difference in *direct emotional support-seeking*,* direct instrumental support-seeking*, or *no support wanted*. Participants recruited via Prolific reported higher indirect support-seeking than those recruited via social media, *t*(463.43) = −3.04, *p* =.002, d = 0.28, 95% CI [−0.46, −0.10], but this effect size was small.

### Discussion

Study 2 revealed 17 items that loaded highly onto a four**-**factor model of the Romantic Support-Seeking (RoSS) scale. These factors aligned with the findings of Study 1, distinguishing between direct, indirect, emotional, and instrumental support-seeking, in line with previous research (Collins & Feeney, 2000; Rife et al., 2016; Verhofstadt, 2007). In addition, the inclusion of *no support wanted* as a subscale can differentiate individuals who do not wish to seek support, but may still expect to receive it, from those who specifically prefer to handle distress independently. The emergence of this subscale is in line with the findings of Study 1 and previous research (see Mikulincer & Shaver, 2016), confirming that some individuals do not seek support either due to fear of rejection or the belief that support should be provided without needing to be requested, while others actively choose to cope alone. These are conceptually distinct factors.

However, relying solely on statistical criteria in item reduction can undermine the conceptual model on which the scale was developed (Goetz et al., 2013; Stanton et al., 2006). Therefore, we considered the qualitative content-oriented features of the scale and chose to remove *partner just knows* as a subscale – because conceptually, this factor differed from others by reflecting a passive reception of support rather than an active decision to engage, or not engage, in support-seeking. Given that the purpose of this scale is to assess specific support-seeking strategies, this factor would have compromised the coherence and theoretical focus of the scale.

Furthermore, participants recruited via Prolific (*M =* 3.86) reported significantly more *indirect support-seeking* than those recruited via social media (*M =* 3.26), with a small effect size. This could be due to several factors. Despite both groups being anonymous, social media platforms include identifiable information so these participants may have felt less anonymous and resultantly been subject to social desirability bias. Alternatively, Prolific participants may be more aware of their own behavior due to regular participation in surveys, so are more likely to notice their own subtle behaviors and report honestly.

## Study 3

Study 3 aimed to evaluate the model fit of the RoSS using confirmatory factor analysis (CFA) with an independent sample. Additionally, this study examined correlations with related constructs to assess preliminary convergent, divergent, and predictive validity. Specifically, we tested correlations between each subscale of the RoSS with the Stress Communicated by Oneself (SCO) subscale of the Dyadic Coping Inventory (Bodenmann, 2008). The SCO is an established measure of support-seeking and we expected to find a positive correlation between the SCO subscale and the *direct emotional support-seeking* and *direct instrumental support-seeking* subscales of the RoSS. We expected a negative correlation between the SCO subscale and the *no support wanted* subscale of the RoSS.

To establish preliminary discriminant validity, we tested correlations of subscales with attachment avoidance and attachment anxiety. Avoidance is associated with reduced (Mikulincer & Shaver, 2016) or ineffective support-seeking (Collins & Feeney, 2000), but a preference for instrumental support over emotional support (Mikulincer & Florian, 1997). Thus, we expected a negative correlation between attachment avoidance and *direct emotional support seeking* and a positive correlation with *no support wanted*. Contrarily, anxiety is correlated with excessive need for support (for a review, see McLeod et al., 2020), emotional reassurance seeking (Brennan & Carnelley, 1999; Shaver et al., 2005), or indirect support-seeking (Fraley & Shaver, 1998). Therefore, we expected anxiety to be positively correlated with *direct emotional support-seeking* and *indirect support-seeking*. To demonstrate discriminant validity, correlations should be below 0.75 (for a review, see Cheung et al., 2024). We additionally conducted a regression analysis between scale scores and attachment, because this may be a better indicator of discriminant validity (Boateng et al., 2018).

Additionally, we examined whether the subscales correlated with theoretically-relevant constructs as expected, to provide preliminary evidence of the predictive validity of the RoSS subscales. Based on previous research, we expected the subscales *direct emotional support-seeking* and *direct instrumental support-seeking* to be positively associated with individual and relationship outcomes (Collins & Feeney; Hilpert et al., 2018) – specifically, relationship quality, life satisfaction, and sexual satisfaction. We expected *direct emotional support-seeking* and *direct instrumental support-seeking* to be positively associated with perceived support, because direct support-seeking elicits responsive caregiving (Collins & Feeney, 2000). We expected a positive correlation between perceived stress and *no support wanted*, because individuals who do not seek support must manage distress alone. Similarly, we expected a positive correlation between perceived stress with *indirect support-seeking* as this elicits unresponsive caregiving that is positively associated with stress (Williams & Mickelson, 2008).

### Method

#### Participants

Participants were recruited via the institution’s participant pool. They were required to be at least 18 years old and in a romantic relationship lasting at least 6 months. Participants received research credits for their participation. Initially, 374 participants began the survey, but data was removed from participants who did not complete the entire survey (*n =* 19) so a total of 355 participants were included in analysis, aligning with research suggesting a minimum of 300 participants for factor analysis or 5:1 or 10:1 participants per items (for a review, see Boateng et al., 2018).

Participants were students at a UK university, with an average age of 20.04 years (*SD =* 2.60, range 18–48). Participants were primarily living in the UK (96.90%). Most participants identified as White (81.97%), Asian (7.89%), Mixed race (5.35%), or Black (1.97%). Most participants were women (89.30%) or men (9.86%). Participants primarily identified as heterosexual individuals (71.55%), bisexual individuals (21.12%), and lesbian or gay individuals (6.20%). Most participants were in a committed relationship (72.11%), dating (21.12%), cohabiting (6.20%), or married (0.56%). Most relationships were monogamous (99.15%) rather than non-monogamous (0.84%). The average length of relationships was 2.10 years (*SD =* 3.18). The majority did not have children (98.03%).

#### Procedure

The survey was completed on Qualtrics. Participants provided demographic information, then completed the RoSS, measuring *direct emotional support-seeking* (α = 0.80, *M =* 7.77, *SD =* 1.55), *direct instrumental support-seeking* (α = 0.77, *M =* 6.86, *SD =* 1.88), *indirect support-seeking (*α = 0.74, *M =* 3.75, *SD =* 2.03), and *no support wanted* (α = 0.79, *M =* 4.09, *SD =* 2.11). Next, they completed scales measuring attachment, relationship quality, sexual satisfaction, perceived support, perceived stress, life satisfaction, and dyadic coping, in this order.

#### Measures

##### Stress communicated by oneself

We used the Stress Communicated by Oneself (SCO) subscale of the Dyadic Coping Inventory (Bodenmann, 2008). Participants rated four items regarding support-seeking from a partner (α = 0.53, *M =* 2.51, *SD =* 0.044) from 1 (*Very rarely*) to 5 (*Very often*): “I let my partner know that I appreciate his/her practical support, advice, or help”; “I ask my partner to do things for me when I have too much to do”; “I show my partner through my behavior when I am not doing well or when I have problems”; “I tell my partner openly how I feel and that I would appreciate his/her support”). Higher mean scores indicated greater stress communicated by oneself.

##### Attachment orientation

Attachment orientation was measured using the shortened version of the Experience in Close Relationship scale (ECR-12; Lafontaine et al., 2016). This includes twelve Likert-scale items across two six-item subscales: anxiety (e.g., “I worry a fair amount about losing my partner”, *α* = 0.89, *M =* 5.01, *SD =* 2.53) and avoidance (e.g., “I don’t feel comfortable opening up to my partner *α* =.89, *M =* 1.94, *SD =* 1.68). Participants indicated their agreement with each statement from 0 (*Disagree strongly*) to 10 (*Agree strongly*).

##### Relationship quality

One item was taken from each subscale of the Perceived Relationship Quality Components Inventory (Fletcher et al., 2000): *satisfaction*,* commitment*,* intimacy*,* trust*,* passion*, and *love* (*α* = 0.89, *M =* 8.70, *SD =* 1.72). Participants rated their relationship with their partner (e.g., “How much do you love your partner?”) on a scale from 0 (*Not at all*) to 10 (*Extremely*). Higher mean scores indicated greater relationship quality.

##### Sexual satisfaction

Sexual satisfaction was measured with one created item: “How sexually satisfied are you with your relationship?” (*M =* 8.13, *SD =* 2.20) on a scale from 0 (*Not at all*) to 10 (*Extremely*).

##### Perceived support

Four items were created to measure perceived support (*α* = 0.93, *M =* 8.30, *SD =* 1.72; “How emotionally supportive (e.g., providing comfort, encouragement, and/or reassurance) is your partner?”, “How practically supportive (e.g., providing advice and/or assistance) is your partner?”, “How satisfied are you with the amount of support your partner provides?”, and “How satisfied are you with how your partner supports you?”) from 0 (*Not at all*) to 10 (*Extremely*). Higher mean scores indicated greater perceived support.

##### Perceived stress

We used the four-item Perceived Stress Scale (PSS-4; Cohen et al., 1983). Participants rate their thoughts and feelings over the last month (*α* = 0.75, *M =* 2.07, *SD =* 0.83; e.g., “In the last month, how often have you felt that difficulties were piling up so high that you could not overcome them?”) on a scale from 0 (*Never*) to 10 (*Very often*). Higher mean scores indicated greater perceived stress.

##### Life satisfaction

Life satisfaction was measured using the Satisfaction with Life Scale (Diener et al., 1985). Participants rated their agreement with five statements (α = 0.91, *M =* 6.15, *SD =* 2.13; e.g., “In most ways my life is close to ideal”) from 0 (*Not at all*) to 10 (*Extremely*). Higher mean scores indicated greater satisfaction with life.

#### Analytic strategy

To confirm the four**-**factor structure of our scale, we performed a CFA with the 17 items, using lavaan (Rosseel, 2012). We compared this four-factor model to a baseline one-factor model, a two-factor model (direct emotional and direct instrumental; indirect and no support wanted) support-seeking, an alternative two-factor model (direct emotional, direct instrumental, and indirect; no support wanted), and three-factor model (direct emotional and direct instrumental; indirect; no support wanted). Goodness of fit was assessed using Maximum Likelihood chi-squared (*χ*^*2*^), comparative fit index (CFI), root mean square error approximation (RMSEA), and standardised root mean square residual (SRMR). A model with good fit is indicated by RMSEA and SRMR indices below 0.08 (Hu & Bentler, 1998) and CFI indices of at least 0.9 (Bentler & Bonett, 1980). Ideally, the *χ*^*2*^ will be nonsignificant, but this is highly sensitive to sample size so often considered too conservative (e.g., Bentler & Bonett, 1980). Alternatively, *χ*^*2*^/degrees of freedom should be below 3.

### Results

The CFA was conducted using R. The indicators suggested that the four-factor model had a reasonable fit to the data: CFI = 0.91, Tucker-Lewis Index (TLI) *=* 0.87, RMSEA = 0.07, SRMR = 0.06, χ2(113) = 312.18, *p* <.001, *χ*^*2*^/df = 2.76. The one-factor model had poor fit: CFI = 0.52, TLI = 0.45, RMSEA = 0.15, SRMR = 0.13, *χ*^*2*^ (119) = 1124.27, *p* <.001, *χ*^*2*^/df = 9.45. The two-factor adaptive-maladaptive model had poor fit: CFI = 0.57, TLI = 0.50, RMSEA = 0.15, SRMR = 0.16, χ2(118) = 1024.18, *p <*.001, *χ*^*2*^/df = 8.68. The two-factor support-seeking and no support-seeking model had poor fit: CFI = 0.67, TLI = 0.62, RMSEA = 0.13, SRMR = 0.12, χ2(118) = 815.37, *p <*.001, *χ*^*2*^/df = 6.91. The three-factor model had poor fit: CFI = 0.81, TLI = 0.77, RMSEA = 0.10, SRMR = 0.08, χ2(136) = 521.65, *p <*.001, *χ*^*2*^/df = 3.84. Thus, the four-factor model best fit the data. The factor loadings can be found in Table 3. Following Study 3, we removed the stem “when I feel stressed” from the item “I go to my partner for encouragement”, because this overlaps with the scale instructions and prompt so is not necessary.

**Table 3:** Factor loadings obtained in the CFA

Subscale	Item	Unstandardised factor loading	Standardised factor loading	M	SD	Sk	Ku
Direct emotional support-seeking	I seek reassurance or comfort from my partner.	1.06	0.80	8.22	1.87	−1.38	5.06
	I ask for physical comfort, such as hugs, from my partner.	1.00	0.66	8.31	2.13	−1.66	5.77
	I seek verbal reassurance from my partner rather than advice (e.g., “everything will be okay”).	0.90	0.50	6.75	2.54	−0.77	3.10
	I go to my partner for encouragement.	1.02	0.76	7.74	1.91	−1.01	4.02
	I seek sympathy and understanding from my partner.	0.91	0.67	7.81	1.92	−0.98	3.96
Direct instrumental support-seeking	I ask to discuss different options with my partner.	0.55	0.48	6.58	2.47	−0.58	2.72
	I discuss the pros and cons about the situation with my partner.	0.55	0.51	7.22	2.93	−0.89	3.37
	I ask my partner for advice directly.	1.00	0.85	6.96	2.50	−0.80	2.88
	I ask my partner for help directly.	0.99	0.85	6.68	2.48	−0.61	2.69
Indirect support-seeking	I complain to my partner that they haven’t been supportive toward me.	1.21	0.73	3.35	2.65	0.65	2.56
	I shout or yell at my partner if they are not supporting me.	1.02	0.68	1.94	2.40	1.34	4.00
	I sulk if I feel unsupported by my partner.	1.16	0.64	4.90	2.91	−0.14	1.90
	I provide non-verbal cues such as huffing and puffing.	1.00	0.55	4.87	2.89	−0.12	1.94
No support wanted	I prefer to do things myself rather than ask my partner for support.	1.00	0.82	4.62	2.62	0.07	2.21
	I prefer to solve my problems by myself rather than ask my partner.	1.03	0.83	4.72	2.65	0.05	2.10
	I don’t tell my partner if I’m stressed unless it’s absolutely necessary.	0.79	0.62	3.67	2.77	0.58	2.34
	I have a hard time accepting support from my partner even if they offer.	0.73	0.56	3.37	2.79	0.67	2.39

**Fig. 1:**
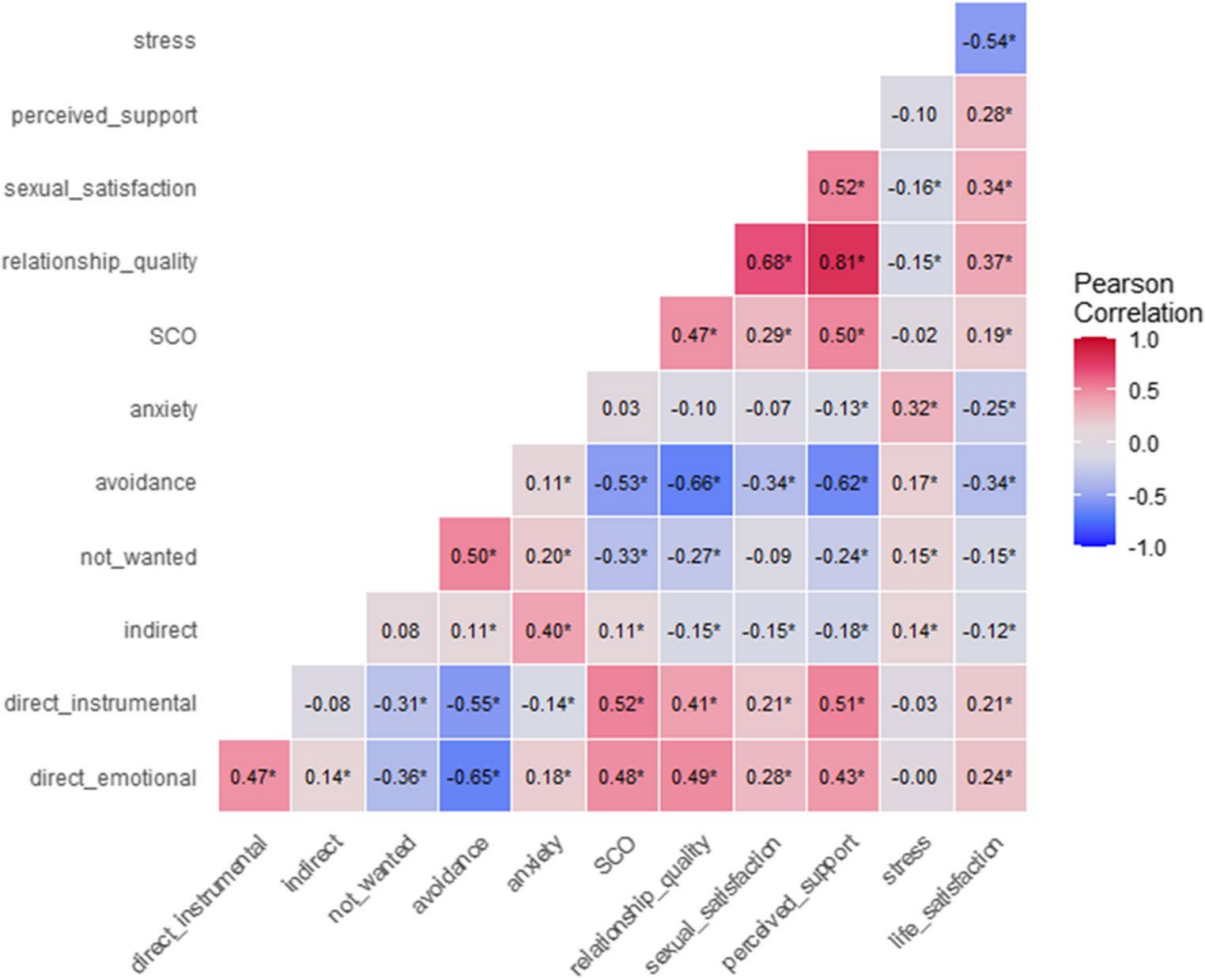
Correlations between subscales and validation measures. Significant correlations are indicated by an asterisk. SCO = Stress Communicated by Oneself – subscale from the Dyadic Coping Inventory (Bodenmann et al., 2008)

We examined the scale validity by correlating subscales with related constructs (see Fig. 1). The findings indicate high convergent validity: Stress Communicated by Oneself (SCO) was positively correlated with *direct emotional support-seeking* and *direct instrumental support-seeking*, whereas SCO was negatively correlated with *no support wanted*. SCO did not correlate with *indirect support-seeking*.

The findings also indicate good discriminant validity through correlation and regression analyses. Attachment avoidance was negatively correlated with direct emotional and instrumental support-seeking, positively correlated with no support wanted, and not correlated with indirect support-seeking. Avoidance negatively predicted *direct emotional support-seeking* (*B* = − 0.63, *SE =* 0.04, *t =* −17.69, *p <*.001) and *direct instrumental support-seeking* (*B* = − 0.61, *SE =* 0.05, *t =* −12.15, *p <*.001). Avoidance positively predicted *no support wanted* (*B* = 0.61, *SE =* 0.04, *t =* 10.67, *p <*.001) and did not predict *indirect support-seeking.* Attachment anxiety was positively correlated with *direct emotional support-seeking*,* indirect support-seeking*, and *no support wanted*, and negatively correlated with *direct instrumental support-seeking*. Anxiety positively predicted *direct emotional support-seeking* (*B* = 0.16, *SE =* 0.02, *t =* 6.81, *p <*.001), *indirect support-seeking* (*B* = 0.31, *SE =* 0.04, *t =* 7.90, *p <*.001), and *no support wanted* (*B* = 0.12, *SE =* 0.04, *t =* 3.19, *p =*.002). Each correlation was below 0.75 (range 0.55–0.66), thus did not demonstrate any issues with discriminant validity.

Lastly, our scale subscales were associated with theoretically related constructs in ways consistent with existing theory, showed good preliminary evidence of predictive validity. Both *direct emotional support-seeking* and *direct instrumental support-seeking* positively correlated with relationship quality, sexual satisfaction, perceived support, and life satisfaction. Both *indirect support-seeking* and *no support wanted* negatively correlated with relationship quality, perceived support, and life satisfaction. *Indirect support-seeking* was negatively correlated with sexual satisfaction, but *no support wanted* was uncorrelated. Neither *direct emotional support-seeking* nor *direct instrumental support-seeking* were correlated with perceived stress. Perceived stress was positively correlated with *indirect support-seeking* and *no support wanted*.

### Discussion

Study 3 demonstrates that the four-factor model of the RoSS is appropriate with acceptable fit. The alphas indicate good reliability (Tavakol & Dennick, 2011) for each subscale, ranging from 0.74 to 0.80. Contrastingly, the reliability alpha for SCO was low at 0.53 – comparable to Shujja et al. (*α =* 0.41; 2020) and Wendołowska et al. (*α =* 0.57; 2022) - highlighting the requirement for a reliable self-report measure of support-seeking. Additionally, Study 3 again reinforces the conceptual distinction between not wishing to seek support – indicated by low scores on the support-seeking subscales - from those who actively prefer to cope independently. This aligns with previous research and Study 1, building on Study 2 to reinforce the distinctiveness of this factor.

Study 3 assessed the validity of the RoSS by correlating the subscales with related measures. Convergent validity because *direct emotional support-seeking* and *direct instrumental support-seeking* positively correlated with SCO, while *no support wanted* negatively correlated with SCO – aligning with expectations. Divergent validity was demonstrated because correlations with attachment avoidance and anxiety aligned with expectations. Avoidance was negatively correlated with *direct emotional support-seeking* and *direct instrumental support-seeking*, and positively correlated with *no support wanted*. This aligns with findings that avoidantly attached individuals avoid support-seeking (Mikulincer & Shaver, 2016). Attachment anxiety was positively correlated with *direct emotional support-seeking*, reflecting the tendency for anxiously attached individuals to seek excessive emotional reassurance (Brennan & Carnelley, 1999; Shaver et al., 2005). Anxiety was positively correlated with *indirect support-seeking*, aligning with current literature (Mikulincer & Shaver, 2016). Anxiety was positively correlated with *no support wanted*, reflecting the hesitancy of anxiously attached individuals to seek support if they doubt its availability (Mikulincer & Shaver, 2016).

Additionally, subscales were associated with theoretically-relevant constructs as expected, showing preliminary evidence of predictive validity *Direct emotional support-seeking* and *direct instrumental support-seeking* positively correlated with relationship quality, sexual satisfaction, perceived support, and life satisfaction **-** aligned with research suggesting direct support-seeking is associated with relational and personal wellbeing (Collins & Feeney, 2000; Hilpert et al., 2018). Conversely, *indirect support-seeking* and *no support wanted* correlated negatively with relationship quality, perceived support, and life satisfaction. *Indirect support-seeking* was negatively correlated with sexual satisfaction. These findings align with evidence that indirect support-seeking elicits ineffective caregiving that decreases relational and personal wellbeing (Don et al., 2019; Williams & Mickelson, 2008), whereas absence of support-seeking does not elicit any caregiving. Neither *direct emotional support-seeking* nor *direct instrumental support-seeking* were correlated with perceived stress, which is somewhat surprising because individuals seek support during distress (Mikulincer & Shaver, 2016), but could be due to support-seeking eliciting caregiving that buffers against stress (Hilpert et al., 2018). Stress was positively correlated with *indirect support-seeking* – supporting previous research suggesting indirect support-seeking elicits unresponsive caregiving that is associated with stress (Williams & Mickelson, 2008). Stress was positively correlated with *no support wanted* **-** which is expected considering these individuals manage distress alone. Overall, the RoSS subscales predict relevant constructs as anticipated based on prior research, highlighting the scale’s validity in assessing support-seeking within romantic relationships.

## General discussion

Support-seekers have traditionally been viewed as passive participants in receiving support (Feeney & Collins, 2015a) rather than active agents eliciting support from their partner. Previously, no scale captured the varied types of support-seeking and coping strategies used by couples. Therefore, we aimed to develop one scale encompassing direct, indirect, emotional, and instrumental support-seeking, as well as capturing those who prefer to cope independently. The RoSS consists of four distinct factors measuring *direct emotional support-seeking*,* direct instrumental support-seeking*,* indirect support-seeking*, and *no support wanted*.

### Findings

The RoSS benefits from empirically and theoretically-driven generation of items. Initial items were based on previous literature, qualitative interviews (Francois-Walcott et al., 2024), and open-ended responses from Study 1. Within Study 1, we identified four themes (*direct*,* indirect*,* relationship-focused*,* no support-seeking*) and 11 subthemes regarding support-seeking behaviors. Items were pooled together and created based on previous literature distinguishing direct, indirect, emotional, and instrumental support-seeking, and preferring not to seek support. Items were administered in Study 2, with 17-items and four subscales retained: *direct emotional support-seeking*,* direct instrumental support-seeking*,* indirect support-seeking*, and *no support wanted*. Finally, the CFA confirmed the four-factor structure and we established preliminary convergent, divergent, and predictive validity in Study 3.

Specifically, the RoSS subscales correlated with SCO as expected - demonstrating convergent validity. However, the SCO showed very low reliability, which complicates interpretation as correlations with a measure of such poor internal consistency are difficult to evaluate. It is possible that the magnitude of these correlations may be attenuated by the low reliability of the SCO measure. The results should be interpreted cautiously with this in mind.

Nevertheless, the RoSS subscales additionally correlated with other theoretically-related constructs in line with expectations. Each subscale correlated with attachment anxiety and avoidance to demonstrate divergent validity, aligning with previous research showing avoidant individuals do not seek support (Collins & Feeney, 2000; Mikulincer & Shaver, 2016), whereas anxious individuals engage in emotional support-seeking (Brennan & Carnelley, 1999; Shaver et al., 2005), indirect support-seeking (Fraley & Shaver, 1998), or no support-seeking (Mikulincer & Shaver, 2016). Lastly, the RoSS shows preliminary evidence of predictive validity as direct support-seeking positively correlated with individual and relational wellbeing, aligning with previous research (Hilpert et al., 2018). Indirect support-seeking was negatively associated with relationship satisfaction, aligning with previous findings (Don et al., 2013, 2019; Williams & Mickelson, 2008).

The RoSS emerges as a reliable and valid measure of romantic support-seeking. Each subscale is short enough to prevent participant fatigue but long enough to gain depth. Subscales are unique and hold internal reliability – especially compared to existing measures of support-seeking, like the SCO, which demonstrated low reliability.

### Implications for theory, research, and practice

This research holds theoretical implications. Firstly, we statistically demonstrated distinct categories within support-seeking. In line with the findings of Study 1 and previous literature (Mikulincer & Shaver, 2016), the inclusion of no support wanted as a subscale means the RoSS can distinguish between individuals who do not want to seek support from those who do not want to receive support. Those who score highly on this subscale actively “prefer to do things [by themselves]” and “prefer to solve [their] own problems”. This reflects a specific choice to not seek or receive support. In contrast, some individuals may desire support but be reluctant to seek support – potentially due to the perceived costs (for a review, see Wang et al., 2023). High & Crowley (2018) found that some individuals desire support but hesitate to seek it, leading to a gap between their need for support and their actual support-seeking behavior. This would instead by reflected by low scores on other subscales. This distinction was clearly demonstrated across all three studies and is crucial for understanding variations in support-seeking behavior and the underlying motivations behind them. Additionally, whilst direct support-seeking was separated into emotional and instrumental support-seeking, no such distinction exists within indirect support-seeking - likely because requests for support are not explicit so it is ambiguous as to whether support is needed and what type of support is needed. While we did identify a fifth factor, *partner just knows*, this did not coherently fit within the active support-seeking measure and so was removed. However, this does have theoretical implications because it reflects an implicit relational dynamic in which partners recognize and address needs without explicit communication.

The RoSS has implications for research, providing a reliable, valid measure of support-seeking. These four separate subscales can be administered together or alone to fit the research scope but should not be considered as one overall factor of support-seeking. Wide use of the RoSS would ease comparison of findings across studies into romantic support-seeking in future research.

The RoSS holds practical implications for counselling or clinical psychologists. Practitioners can use the RoSS to identify ineffective support-seeking strategies and encourage clients to be direct to find more adaptive methods of seeking support. Directly requesting support is linked to thriving relationships (Feeney & Collins, 2015b) and is associated with many positive outcomes (Collins & Feeney, 2000; Hilpert et al., 2018). Moreover, direct support-seeking is more likely to elicit the preferred type of support, leading to beneficial outcomes (Cutrona et al., 2007). Conversely, the highest loading items for indirect support-seeking generally refer to negative communication patterns that can be damaging to relationships, aligning with previous research (e.g., Collins & Feeney, 2000). Psychologists should address these behavior patterns to ensure couples communicate and seek support effectively, to benefit their individual and relational wellbeing.

### Limitations and future directions

There are notable limitations of this research to be considered when utilising the RoSS. Firstly, all three studies used cross-sectional data with individuals, limiting causal implications. Longitudinal research is needed to assess predictive validity to establish the explanatory power of the subscales over relevant outcome variables. Dyadic data is needed to understand the dynamic and reciprocal nature of support behaviors (for a review, see McLeod et al., 2020). Additionally, the scale does not capture the excessive support-seeking demonstrated by anxiously attached individuals (Brennan & Carnelley, 1999; Shaver et al., 2005) as we did not measure frequency.

Furthermore, our samples were predominantly White, heterosexual participants. Research has demonstrated individuals perceiving stigma use indirect support-seeking due to fear of rejection, resulting in unsupportive network responses (Williams & Mickelson, 2008). Likewise, research has demonstrated cultural differences in support-seeking – those from collectivist cultures may be less willing to seek support (for a review, see Wang et al., 2023). Future research should validate the RoSS across diverse demographics. The samples were also predominantly female, but there is evidence that women may seek more support than men (for a review, see Wang et al., 2023) – thus, the findings of this research primarily reflect women’s experiences. In addition, both Study 1 and Study 3 used student samples with a low mean age (19.40 and 20.04 respectively), so these results reflect the experiences of individuals in emerging adulthood but may not be representative of older samples. This may limit comparison between studies, because Study 2 had a broader age range. Future studies should recruit more balanced samples and explore these differences and examine whether the RoSS functions equivalently across age ranges. The limitations of this sample means that further work is needed to establish whether the scale performs similarly in more diverse populations. While the current findings offer strong initial evidence for the RoSS in young, White, female samples, the extent to which the scale applies to other demographic groups is unclear and needs to be addressed in future research.

Future research should also measure test-retest reliability. This would be an important step to establish temporal stability and ensure that the scale yields reliable results across multiple time points. Although, support-seeking may fluctuate over time because distress, need for support, and partner availability do not remain consistent – thus, the same results may not be demonstrated within the same individuals over time. Research should assess correlations of self-reported scores with other measures, like diaries or observation, to ensure validity beyond shared method variance. The use of a single-item measure of sexual satisfaction is also limited, so further research should use a more robust measure to assess the correlations identified in this research.

Lastly, future research may seek to validate this scale in other contexts. The RoSS could be validated outside of romantic relationships, to be used in other relational contexts – this may be helpful to measure the distinct types of support-seeking within one scale for other relationships, including friendships or familial relationships. This could also allow for comparison of support-seeking behaviors between romantic relationships and other relationships. Furthermore, researchers could validate the scale in other languages and other cultural contexts to allow for more widespread use of this measure, which would aid comparison across studies and between samples.

### Conclusion

Overall, this research provides an empirical and theoretically-driven measure that encompasses differing types of romantic support-seeking across validated subscales. This addresses a gap in the literature because no previous scale had assessed direct, indirect, emotional, and instrumental support from partners within one measure. Thus, the RoSS will be useful for researchers to measure distinct types of romantic support seeking within one reliable, valid scale.

## Supplementary Information

Below is the link to the electronic supplementary material.

Supplementary File 1 (DOCX 16.3 KB)

## Data Availability

All data and analysis have been made publicly available at Open Science Framework: https://osf.io/rv56c/?view_only=83c4a6489a2b46698ed68a71ec230fd7.
