# Supplementary material for: Help! I need somebody: Development and validation of the Romantic Support-Seeking (RoSS) scale
Source: Curr Psychol. 2025 Dec 22;45(1):57. doi: 10.1007/s12144-025-08749-0 (PMC12722480; doi:10.1007/s12144-025-08749-0)
Supplement: Supplementary file 1 — (DOCX 16.3 KB) [file 12144_2025_8749_MOESM1_ESM.docx]

**Table S1**

*Descriptive Statistics of Subscales by Gender in Study 1.*

|  | Overall | | Female | | Male | | Identify in another way | |
| --- | --- | --- | --- | --- | --- | --- | --- | --- |
|  | *M* | *SD* | *M* | *SD* | *M* | *SD* | *M* | *SD* |
| Direct emotional support-seeking | 7. 15 | 1.88 | 7.48 | 1.81 | 6.51 | 1.88 | 7.29 | 0.81 |
| Direct instrumental support-seeking | 7.06 | 2.04 | 7.26 | 1.98 | 6.64 | 2.13 | 7.61 | 1.34 |
| Indirect support-seeking | 3.55 | 2.18 | 3.64 | 2.23 | 3.41 | 2.05 | 2.75 | 2.59 |
| No support wanted | 4.21 | 2.11 | 3.92 | 2.07 | 4.79 | 2.09 | 3.93 | 1.99 |

**Table S2**

*Descriptive Statistics of Subscales by Gender in Study 2.*

|  | Overall | | Female | | Male | | Identify in another way / prefer not to say | |
| --- | --- | --- | --- | --- | --- | --- | --- | --- |
|  | *M* | *SD* | *M* | *SD* | *M* | *SD* | *M* | *SD* |
| Direct emotional support-seeking | 7.76 | 1.55 | 7.90 | 1.43 | 6.41 | 1.98 | 8.47 | 0.95 |
| Direct instrumental support-seeking | 6. 85 | 1.88 | 6.87 | 1.91 | 6.59 | 1.72 | 7.67 | 1.26 |
| Indirect support-seeking | 5.20 | 2.39 | 3.81 | 2.08 | 3.35 | 1.51 | 2.92 | 2.77 |
| No support wanted | 4.11 | 2.12 | 4.01 | 2.11 | 5.06 | 2.07 | 3.00 | 0.25 |
